# A Study of Graduate Students’ Achievement Motivation, Active Learning, and Active Confidence Based on Relevant Research

**DOI:** 10.3389/fpsyg.2022.915770

**Published:** 2022-06-10

**Authors:** Jen-Chia Chang, Yu-Tai Wu, Jhen-Ni Ye

**Affiliations:** ^1^Graduate Institute of Technological and Vocational Education, National Taipei University of Technology, Taipei City, Taiwan; ^2^Office of Physical Education, Soochow University, Taipei City, Taiwan

**Keywords:** academic confidence, active learning, achievement motivation, graduate student, University of Science and Technology

## Abstract

Graduate students’ failure to graduate is of great concern, with the failure to graduate due to the dissertation being the most influential factor. However, there are many factors that influence the writing of a dissertation, and research on these factors that influence graduate students’ learning through emotion and cognition is still quite rare. A review of past research revealed that the main factor causing graduate students to drop out midway is not completing their thesis, followed by factors including insufficient achievement motivation, lack of learning strategy, and low confidence. The graduation rate of graduate students has been emphasized by the academic community; therefore, this study investigated the correlation between graduate students’ achievement motivation, active learning, and academic confidence in writing research. The study invited graduated students from two universities of science and technology situated in the northern region of Taiwan to complete the questionnaire. In this study, valid data for validation analysis were collected from 173 respondents, and the results showed that achievement motivation positively influenced active learning (higher-order learning, integrative learning, reflective learning) and that active learning (higher-order learning, integrative learning, reflective learning) positively influenced academic confidence. From the above findings, it can be seen that to help graduate students from University of Science and Technology to effectively complete their graduate studies, students should develop good motivation to adopt active learning strategies to enhance their academic self-confidence.

## Introduction

In Taiwan, the delayed graduation of graduate students has become an important educational issue of social concern (Ho et al., 2020). Gardner (2009) found that the reasons for the low graduation rate of doctoral students include being unable to complete their degree theses, among others. The completion of the degree thesis is an important milestone and the biggest obstacle for graduate students (Blum, 2010). Muszynski (1990) found that graduate students who fail to graduate in time may be uninterested in the research topic, have low academic confidence, or have too many research papers to complete. Spaulding and Rockinson-Szapkiw (2012) interviewed 76 doctoral graduates and found that motivation, persistence factors, and completion strategies were necessary to complete their dissertations.

However, Pulford et al. (2018) found that people must have motivation before they are willing to put in the effort and persevere. Usta (2017) noted that there are significant mutual influences among an individual’s learning achievement, motivation, and confidence. Belshaw et al. (2020) found that learners who use passive learning not only have lower learning efficiency and less comprehension, but their comprehension level is also low. It has also been found that learners with higher levels of active learning are more willing to engage in learning and gain more knowledge from it, thereby reducing delayed graduation (Schmidt et al., 2009). Wehrens (2008) suggested that learners with low confidence or feelings of inferiority may have limited learning progress, drop out of school, or have other problems. Academic confidence refers to the student’s belief in the learning task and in achieving the learning goal (Sander and Sander, 2005), which reflects the student’s beliefs and expectations for success in the academic field. Students also prefer to perform activities or tasks in which they feel competent (Bandura, 1977; Eccles et al., 1989).

In addition, when students are able to succeed academically, it can also be attributed to their motivation and willingness to put in the effort, which in turn brings motivation for learning and academic confidence, and encourages them to take action and persevere (Pulford et al., 2018). Chemers et al. (2001) found that students’ lack of academic confidence is inextricably linked to their expectation of success and has a significant effect on academic performance, as academic confidence controls students’ desire to learn. If students’ academic confidence is low, it will affect their desire to learn. If they do not have sufficient academic confidence, they may not be eager to learn and may not continue their studies (Ireson and Hallam, 2009). Sander and de la Fuente (2020) found that academic confidence helps students to effectively acquire learning strategies and skills. In addition, de la Fuente et al. (2013) found an interdependent effect between academic confidence, learning methods, and achievement in a study of 2,429 psychology students. Based on this, this study validated the control-value theory of achievement emotion (CVTAE) according to Pekrun (2006).

Regarding CVTAE, Pekrun (2006) mentioned that learners’ assessment of control and value directly predicts their emotional responses, with control referring to the learners’ actions and beliefs about learning tasks (e.g., self-efficacy and attribution) and value referring to the importance learners place on learning tasks and outcomes (Pekrun et al., 2011). When learners attach more importance to learning tasks, good outcomes are achieved (Pekrun, 2006). Garn et al. (2017) found that learners’ perceptions of academic achievement can enhance or reduce the positive or negative outcomes faced in the learning environment. This study used the CVTAE perspective to analyze the correlation between graduate students’ academic achievement motivation, active learning, and academic confidence. It was hoped that the results of this study could be used to help improve the academic confidence of graduate students.

### Achievement Motivation

McClelland (1961) rationalize the motivation of achievement and divide it into three demands (1) achievement, (2) connection, and (3) power. Pintrich and Schunk (1996) defined motivation as the process by which an individual is motivated and sustained in order to achieve a goal, thereby laying an important foundation for accomplishing the goal (e.g., planning, learning, and decision-making). Bigge and Hunt (1980) defined achievement motivation as the motivation to perform and actively work toward a goal without interruption, and gain a sense of accomplishment in the process. Individuals who set a goal they want to achieve will take action to achieve it (Linnenbrink, 2005). Therefore, achievement motivation is also considered as a source of motivation to influence or maintain behavior (Reeve, 2009). The motivation for achievement in this study refers to the graduate student’s motivation for wanting to complete a degree thesis.

Achievement motivation is a subjective value, as well as a psychological drive that helps individuals achieve their goals (Singh, 2011). Urdan and Kaplan (2020) found that each scholar’s definition of achievement motivation is somewhat different; however, achievement motivation is inextricably linked to learning outcomes, emotions, and strategies. Research has found that achievement motivation has the ability to predict academic ability and task success (Liao et al., 2012). In addition, Saiti et al. (2017) suggested that graduate student learning goals and motivation are inextricably linked to individual engagement in learning tasks to meet personal needs and expectations.

### Active Learning

Bonwell and Eison (1991) noted that active learning occurs when an individual takes the initiative to perform a task and thinks about why they are doing it and that active learning is one of the processes of learning that requires learners to learn independently or in groups (Singer et al., 2012). According to previous scholars, active learning is based on three components (1) higher-order learning: learners focus on the exchange of information and knowledge, (2) integrative learning: learners learn from experience, and (3) reflective learning: learners reflect on whether they have learned after the course (Fink, 2003). Matsushita (2018) suggested that active learning is about driving learners to act and to reflect on learning through action. Graduate students are expected to develop independent research knowledge, skills, and experiences before writing their theses (Davis et al., 2017). Active learning emphasizes learners’ deep learning, understanding, and engagement in the learning process (Matsushita, 2018), and active learning expects learners to think, analyze, discuss, and make decisions with their peers as part of an active learning process (Freeman et al., 2014). Active learning in this study refers to the ability of graduate students to understand the learning content completely, to apply their knowledge, and to learn from their mistakes.

In addition, Budsankom et al. (2015) showed that learners’ psychology is a direct and effective influence on higher-order learning. However, Robertson and Howells (2008) argue that integrative learning allows learners to “learn by doing” during the experiential process, which can deepen learners’ memory and practical skills. In addition, Hwang et al. (2014) showed that integrative learning not only allows learners to think deeply about how to solve problems during the learning process, but also to make a reflective search for what they did not do well when their performance was not as expected. Lucas (2001) pointed out that the learner’s motivation affects the learning strategy to be used in learning. Conversely, if learners have low achievement motivation, they will choose to complete their assignments at a lower standard (Davidson, 2002). Research has found that achievement motivation has a positive effect on active learning (Everaert et al., 2017). Therefore, the hypotheses related to the interaction between achievement motivation and active learning were as follows:

*H_1_*: Achievement motivation significantly and positively affects higher-order learning.

*H_2_*: Achievement motivation significantly and positively affects integrative learning.

*H_3_*: Achievement motivation significantly and positively affects reflective learning.

### Academic Confidence

Rosenberg (1965) noted that confidence is an individual’s evaluation of self and that academic confidence is an important competency in the academic world (Adnan et al., 2011). Academic confidence refers to learners’ beliefs about performing tasks and achieving academic goals (Sander and Sander, 2005), which reflects the learners’ beliefs or expectations about success in the field of study. Wehrens (2008) indicated that when learners’ self-confidence is low, it leads to poor performance in learning outcomes, and Bénabou and Tirole (2002) showed that self-confidence has a strong influence on learning performance. Learners usually choose to perform tasks they believe they can accomplish (Bandura, 1977; Eccles et al., 1989), Basturkmen et al. (2014) found that a growing number of graduate students are joining research programs, and found that graduate students need support and encouragement in their writing. For graduate students, academic research, knowledge, skills, competencies, and practices are a serious challenge (Lee and Aitchison, 2009). Aithal and Kumar (2018) suggested that the application of knowledge is skill-dependent and that the application of skills requires confidence. Academic self-confidence in this study refers to the ability of graduate students to find their own research topics and to complete them.

In addition, Retnowati et al. (2018) showed that one of the reasons learners choose to avoid problems with Higher-order Thinking skills is because of low self-confidence and the belief that they cannot achieve the task. Di Francesca (2020) pointed out that engaged learners in active learning environments may build confidence, and some scholars believe that active learning can enhance learners’ responsiveness, confidence, and motivation. (Robinson, 2017; Sibona and Pourrezajourshari, 2018). Research suggests that active learning has an important impact on the development of academic confidence (O'Flaherty and Costabile, 2020). Pekrun (2006) proposed that CVTAE defines learners’ emotions as a regulatory process and that emotions can regulate individuals’ cognition, motivation, etc. Whereas good emotions stimulate individual behavior, bad emotions reduce individual behavior (Pekrun, 2006; Pekrun et al., 2009). Self-confidence is a good source of emotion for the learner, which means that self-confidence and behavior are inextricably linked. Therefore, the hypotheses related to the interaction between active learning and academic confidence were as follows:

*H_4_*: Higher-order learning significantly and positively affects academic confidence.

*H_5_*: Integrative learning significantly and positively affects academic confidence.

*H_6_*: Reflective learning significantly and positively affects academic confidence.

## Materials and Methods

### Research Framework

**Figure 1:**
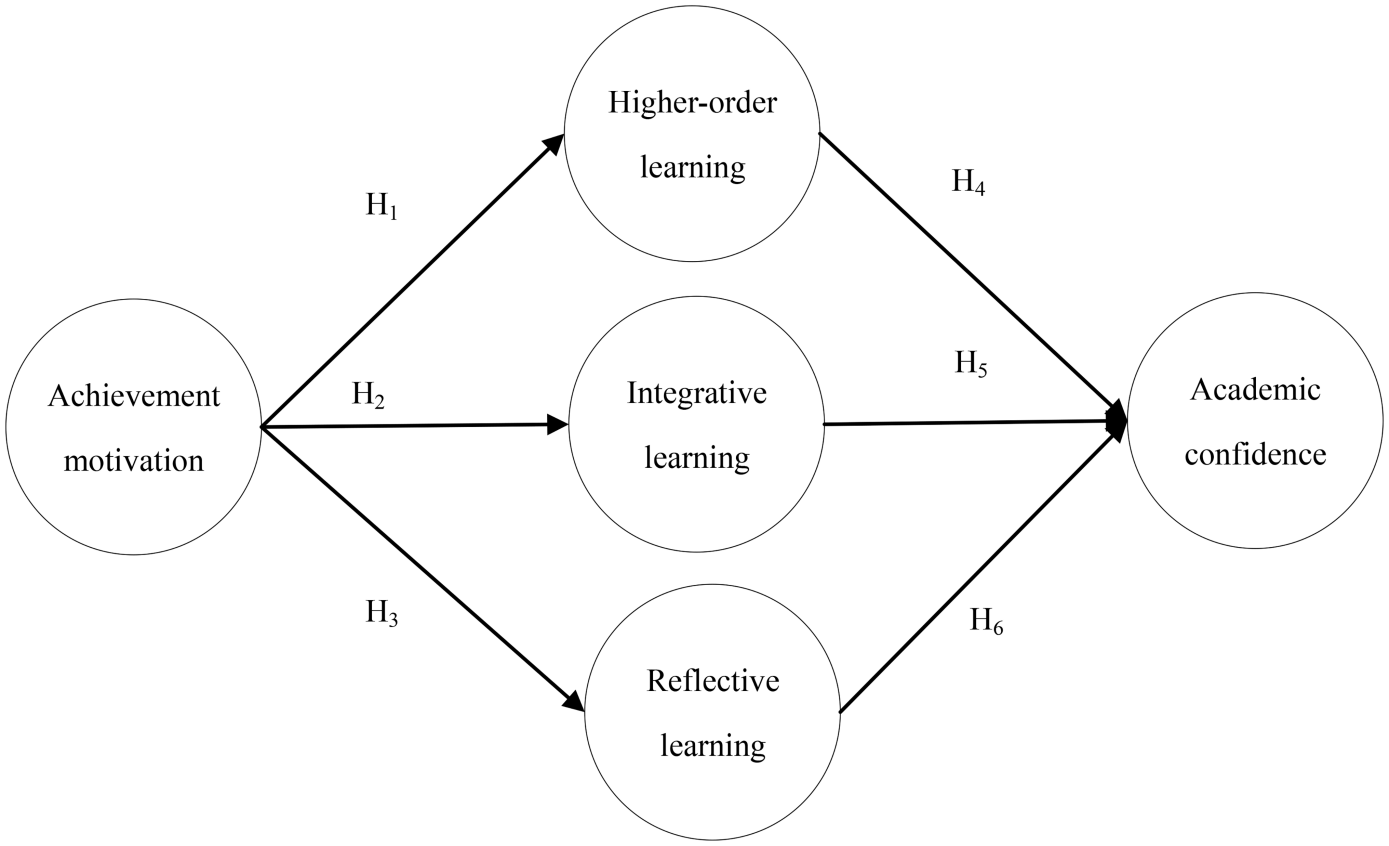
Research model.

Pekrun (2006) and Pekrun (2009) proposed that CVTAE theory can be used to observe whether learners’ beliefs affect their mobility and that when learners judge that a learning task is accomplishable, the next step will be to take action to achieve the goal. Therefore, based on the above literature, we proposed an initial model to investigate the relationship between achievement motivation, active learning, and academic confidence, as shown in Figure 1.

### Procedure

This study was conducted using online questionnaires with purposive sampling, and the questionnaires were collected from November 10, 2019 to December 5, 2019 from graduate students enrolled in universities of science and technology in Taipei City and New Taipei City.

### Participants

There were 205 participants in this study. After 32 invalid samples were deleted, 173 valid samples were collected, indicating a questionnaire recovery rate of 84.39%, as shown in Table 1.

**Table 1:** Basic information.

**Variables**	**Content**
Gender	male: 60 (34.7%) Female: 113 (65.3%)
The Institute studied at	Public Schools: 136 (78.6%) Private Schools: 37 (21.4%)
The academic degree	Master’s: 156 (90.2%) Doctorate: 17 (9.8%)
Research Areas	Education: 57 (32.9%) Arts and Humanities: 23 (13.3%) Technology and Engineering: 24 (13.9%) Business: 38 (22.0%) Medical: 3 (1.7%) Biological and Scientific: 8 (4.6%) Other: 20 (11.6%)

### Measurement

The questionnaire in this study was adapted from a scale developed by a related researcher and was divided into the three components of achievement motivation, active learning, and academic confidence. The results were assessed using a Likert 5-point scale (with answers ranging from 1: *strongly disagree* to 5: *strongly agree*).

#### Achievement Motivation

McClelland theorized achievement motivation in 1961 and identified three different needs for it: (1) the need for achievement; (2) the need for connection; and (3) the need for power (McClelland, 1961). Linnenbrink (2005) noted that achievement goals motivate individuals to engage in task behavior. Some scholars believe that motivation can influence individual behavior, generate interest, or serve as a sustaining force (Reeve, 2009). Pintrich (2003) suggested that motivation is inextricably linked to academic behavior. Hong et al.’s (2017) “Measuring intrinsic motivation of Chinese learning” was adopted to measure participants’ perceptions of their achievement motivation.

#### Active Learning

In Bonwell and Eison (1991) defined active learning strategies as learners actively doing something and thinking about why they are doing it (Bonwell and Eison, 1991). Fink (2003) expanded on the previous foundation to include three additional items: (1) a focus on the learner in the exchange of information and knowledge; (2) allowing learners to actually observe situations; and (3) reflective learning, which involves learners thinking on their own or discussing with others. In addition, Singer et al. (2012) pointed out that active learning is defined as a learning process which requires learners to organize and integrate learning content, either independently or in groups. Therefore, this study revised Entwistle and McCune’s (2004) “Comparison of Scales from Inventories Measuring Study Strategies” to measure the participants’ perceptions of their active learning.

#### Academic Confidence

Bénabou and Tirole (2002) suggested that confidence refers to an individual’s belief in his or her own ability, while Stankov et al. (2012) suggested that confidence is a state in which an individual is certain of the success of a task or behavior. However, insufficient confidence or feelings of inferiority can cause learners to perform poorly in their learning (Wehrens, 2008). Some studies have pointed out that confidence has a motivational effect on learning (Bénabou and Tirole, 2002). Therefore, this study revised Sander (2009) “Academic Behavioural Confidence Scale” to measure the participants’ perceptions of their academic confidence.

### Data Analysis

Structural Equation Modeling (SEM) is commonly used in the fields of psychology, sociology, and education (Teo et al., 2013), and is often used to analyze the correlations between potential variables (Hair et al., 2014). This study used SPSS for the descriptive statistics, Cronbach’s alpha reliability, and external validity, and used AMOS for the model and fitness validation.

## Results and Discussion

### Item Suitability Analysis

The item analysis in this study was conducted using first-order confirmatory factor analysis. According to scholarly recommendations: the *χ*^2^/*df* value should not be greater than 5; the RMSEA should not be greater than 0.1; neither the GFI nor the AGFI should be less than 0.8; and the factor loading (FL) should not be less than 0.5 (Hair et al., 2010; Kenny et al., 2015). As a result, the number of items regarding achievement motivation was reduced from nine to five; higher-order learning was reduced from seven to four; integrative learning was reduced from seven to five; reflective learning was reduced from seven to five; and academic confidence was reduced from seven to four items (Table 2).

**Table 2:** Item analysis by first-order confirmatory factor analysis.

Index	Threshold	Achievement motivation	Higher-order learning	Integrative learning	Reflective learning	Academic confidence
*χ* ^2^	–	13.059	1.255	9.585	2.112	3.364
*df*	–	5	2	5	2	2
*χ*^2^/*df*	< 5	2.612	0.628	1.917	1.056	1.682
RMSEA	<0.1	0.097	0.000	0.073	0.018	0.063
GFI	>0.8	0.972	0.996	0.977	0.994	0.990
AGFI	>0.8	0.916	0.982	0.931	0.971	0.950

### Construct Reliability and Validity Analysis

The reliability of this study was first verified by Cronbach’s α to verify the internal consistency, and then the composite reliability (CR) was used to check the reliability (Hair et al., 2010). In this study, the Cronbach’s α values ranged from 0.789 to 0.903 and the CR values ranged from 0.783 to 0.903, all of which met the criteria suggested by scholars, as shown in Table 3.

**Table 3:** Reliability and validity analysis.

Items	*M*	*SD*	FL	*t* value
**Achievement motivation** *M* = 3.617, *SD* = 0.725, Cronbach’s α = 0.882, CR = 0.875, AVE = 0.585				
1. I am willing to put in extra effort to avoid the research from being abandoned halfway.	3.71	1.010	0.848	15.073
2. No matter how big the obstacles are, I will try to overcome them when conducting research.	3.70	0.843	0.811	11.880
3. I want to be admired through my research performance.	3.57	0.917	0.706	11.217
4. I will try to carry out the research without worrying that the process will be too difficult.	3.50	0.819	0.648	12.845
5. When I encounter a difficult research problem, I will change my approach according to the time and place.	3.61	0.796	0.792	11.625
**Higher-order learning** *M* = 3.646, *SD* = 0.734, Cronbach’s α = 0.843, CR = 0.846, AVE = 0.584				
1. I learn from understanding.	3.78	0.951	0.900	10.808
2. I learn from understanding, not from remembering.	3.71	0.952	0.824	13.587
3. I will look for different ways to solve the problem and make assumptions to recheck them.	3.53	0.804	0.578	10.658
4. When I read, I judge the meaning of the message and apply it to the appropriate context, for example, a conference event.	3.57	0.844	0.717	12.617
**Integrative learning** *M* = 3.697, *SD* = 0.725, Cronbach’s α = 0.903, CR = 0.903, AVE = 0.651				
1. In class discussions, I bring together knowledge, ideas, or concepts from different courses.	3.65	0.798	0.725	9.660
2. I often combine what I have been taught in school with my daily life experience.	3.75	0.898	0.809	11.395
3. When learning new knowledge, I try to link it to my past learning experiences.	3.80	0.925	0.895	11.454
4. I find myself thinking about the commonalities of the different course content.	3.69	0.831	0.802	12.399
5. I try to integrate ideas, information, or experiences into new and more complex explanations and relationships.	3.60	0.813	0.794	14.111
**Reflective learning** *M* = 3.642, *SD* = 0.626, Cronbach’s α = 0.790, CR = 0.789, AVE = 0.485				
1. I think about the problem from a third person’s (his/her) perspective so that I can better understand someone else’s point of view.	3.80	0.826	0.765	9.162
2. I can learn new things from my mistakes and can change the way I understand the problem or concept.	3.51	0.782	0.581	10.842
3. I tried to come up with ideas to build on in the study topic.	3.62	0.780	0.732	11.421
4. I will plan my overall study time to get the most out of my studies.	3.62	0.809	0.694	11.365
**Academic confidence** *M* = 3.474, *SD* = 0.674, Cronbach’s α = 0.789, CR = 0.783, AVE = 0.476				
1. I can easily find innovative research topics and have the confidence to develop a career in academic research.	3.35	0.867	0.629	12.370
2. I am a good self-studier and have the confidence to do well in my research.	3.57	0.857	0.662	11.863
3. I know how to consult my seniors when I encounter difficulties, and I have the confidence to develop my career in academic research.	3.49	0.893	0.698	15.206
4. I like to do all kinds of reflective reasoning, so I have confidence in my academic research career development.	3.49	0.826	0.763	14.286

The average validity of this study was measured by the factor loading (FL) and average variance extracted (AVE). Firstly, it has been recommended by scholars that the FL value should not be lower than 0.5 and that items which are lower than the recommended standard should be deleted (Hair et al., 2010). The FL values for achievement motivation ranged from 0.648 to 0.848; the FL values for higher-order learning ranged from 0.578 to 0.9; the FL values for integrative learning ranged from 0.725 to 0.895; the FL values for reflective learning content ranged from 0.581 to 0.765; and the FL values for academic confidence ranged from 0.629 to 0.763, as shown in Table 3. In addition, scholars believe that the AVE value should not be less than 0.4 (Fraering and Minor, 2006), as shown in Table 3.

Scholars have pointed out that the AVE square root value of each construct should not be lower than the Pearson correlation coefficient value of the remaining constructs, in order for the constructs to have construct discriminant validity (Zainudin, 2015). The correlation value of each construct should not exceed 0.85; if the correlation is higher than 0.85, it will give rise to the concern of multivariate co-linearity (Awang, 2012). The results of this study showed that the constructs had sufficient construct discriminant validity, as shown in Table 4.

**Table 4:** Construct discrimination analysis.

S. No.	Constructs	1	2	3	4	5
1.	Achievement motivation	**(0.765)**				
2.	Higher-order learning	0.549	**(0.764)**			
3.	Integrative learning	0.595	0.655	**(0.807)**		
4.	Reflective learning	0.533	0.594	0.725	**(0.696)**	
5.	Academic confidence	0.524	0.592	0.634	0.616	**(0.670)**

The value on the diagonal is the square root of AVE and the other values are the related coefficients.

### Index Analysis

According to recommendations, *χ*^2^/*df* should not be higher than 5, RMSEA should not be higher than 0.1, PNFI and PGFI should not be lower than 0.5 (Hair et al., 2010), and GFI, AGFI, NFI, NNFI, CFI, IFI, and RFI should not be lower than 0.8 (Abedi et al., 2015). In the present study, the results were as follows: *χ*^2^ = 347.041; *df*. = 203; *χ*^2^/*df*. = 1.710; RMSEA = 0.064; GFI = 0.842; AGFI = 0.803; NFI = 0.853; NNFI = 0.923; CFI = 0.932; IFI = 0.933; RFI = 0.833; PNFI = 0.676; and PGFI = 0.750. The results all met the criteria suggested by scholars, and therefore this study had a good model index.

### Path Analysis

**Figure 2:**
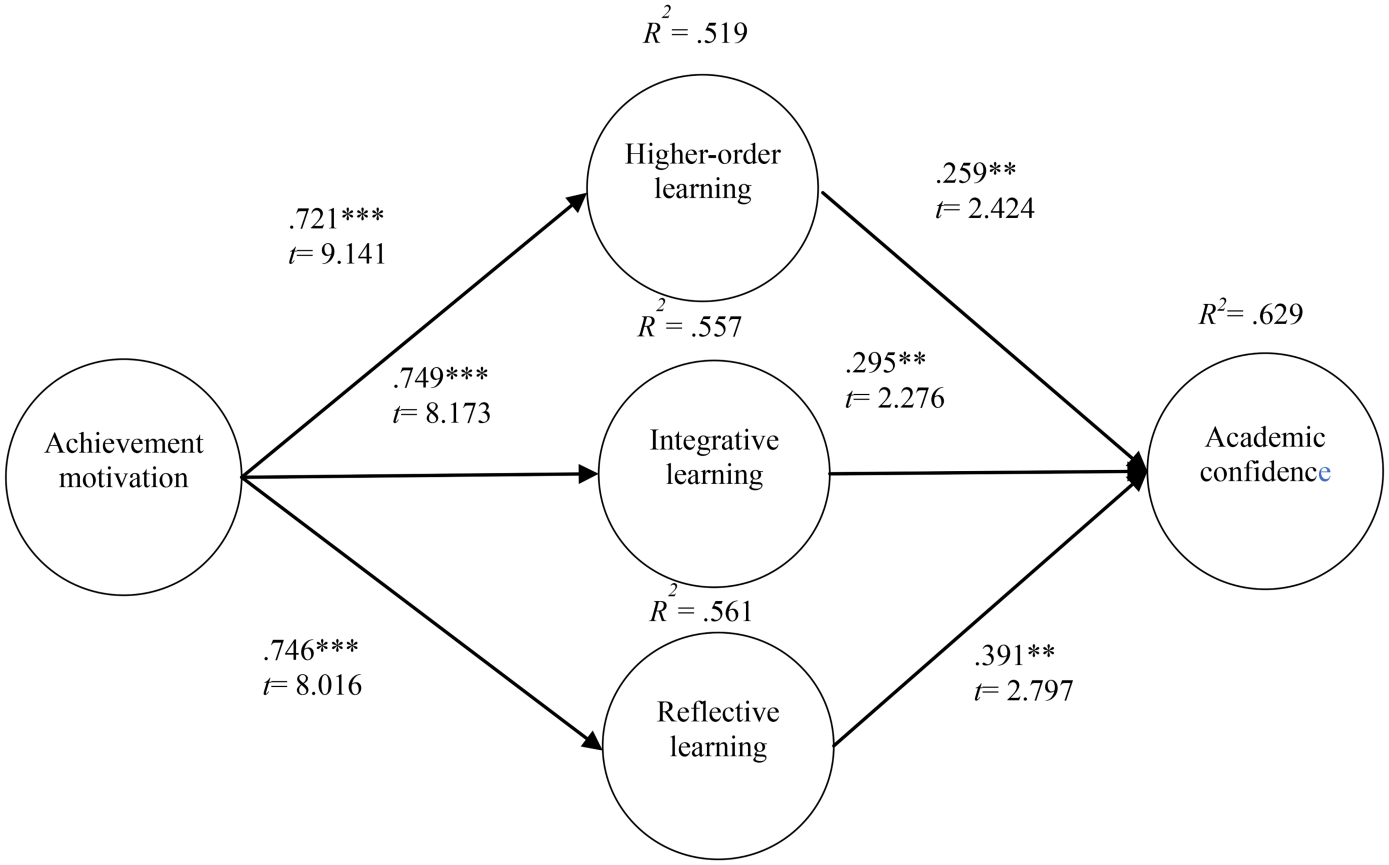
Verification model. ^**^p < 0.01 and ^***^p < 0.001.

The model validation results showed that achievement motivation had a positive effect on higher-order learning (*β* = 0.721, *p* < 0.001, *t* = 9.141), achievement motivation had a positive effect on integrative learning (*β* = 0.749, *p* < 0.001, *t* = 8.173), achievement motivation had a positive effect on reflective learning (*β* = 0.746, *p* < 0.001, *t* = 8.016), higher-order learning had a positive effect on academic confidence (*β* = 0.259, *p* < 0.01, *t* = 2.424), integrative learning had a positive effect on academic confidence (*β* = 0.295, *p* < 0.01, *t* = 2.424), and reflective learning had a positive effect on academic confidence (*β* = 0.391, *p* < 0.01, *t* = 2.797), as shown in Figure 2.

The explanatory power of achievement motivation for higher-order learning was 51.9%; the explanatory power of achievement motivation for integrative learning was 55.7%; the explanatory power of achievement motivation for reflective learning was 56.1%; and the explanatory power of active learning (higher-order learning, integrative learning, and reflective learning) for learning confidence was 62.9%, as shown in Figure 2.

### Discussion

#### Achievement Motivation Positively Influences Academic Active Learning

Entwistle (1998) and Vermunt and Vermetten (2004) stated that teachers encourage learners to adopt active learning because it leads to better learning outcomes, while Skaalvik and Skaalvik (2005) stated that learners’ understanding of learning objectives depends on the level of self-understanding. Ferla et al. (2010) confirmed that learners’ cognitive abilities predict their overall learning strategies, and Everaert et al. (2017) proved that achievement motivation has a positive relationship with active learning. The results of this study validated H_1_, H_2_, and H_3_, and showed that learners’ achievement motivation significantly and positively influences active learning (higher-order learning, integrative learning, and reflective learning), echoing the above study. It could be seen that the higher the learner’s motivation to achieve, the more enthusiastic the learner will be about the learning task and so take the initiative to learn.

#### Active Learning Positively Influences Academic Confidence

Adnan et al. (2011) identified academic confidence as an important competency for scholars in academia, while Sander and Sander (2005) stated that academic confidence gives learners the motivation to perform learning tasks and achieve goals. Libby (1991) confirmed that active learning can increase learning motivation, learning interest, confidence, problem-solving abilities, communication skills, and judgment, while Sibona and Pourrezajourshari (2018) confirmed that active learning can increase confidence. O'Flaherty and Costabile (2020) confirmed that active learning has a positive relationship with academic confidence. This study verified the results of H_4_, H_5_, and H_6_ and confirmed that the active learning (higher-order learning, integrative learning, and reflective learning) of learners has a positive effect on their academic confidence, echoing the above study. This result indicated that the more active learners are in learning, the more their academic confidence will increase.

## Conclusion and Recommendations

### Conclusion and Limitations

Ehrenberg et al. (2009) found that the most important factor for learners to drop out of graduate school is the need to write a dissertation. Graduate students’ motivation, persistence, and strategies used to complete their degree dissertations are essential factors (Spaulding and Rockinson-Szapkiw, 2012). Of course, the learner’s confidence that he or she can complete the research is also an essential factor (Sander and Sander, 2005). In this study, six research hypotheses were formulated using CVTAE theory and were used to determine learners’ mobility, beliefs, and importance of learning (Pekrun, 2006). Through validating the CVTAE framework, a theoretical model of the relationship among achievement motivation, active learning (higher-order learning, integrative learning, and reflective learning) and academic confidence was developed. The results showed that achievement motivation positively influences active learning (higher-order learning, integrative learning, and reflective learning) and that active learning (higher-order learning, integrative learning, and reflective learning) positively influences academic confidence. Therefore, thesis is the biggest factor influencing graduation, and in order to increase the graduation rate, we should improve the motivation of graduate students’ research, and encourage them to submit more journals to train their ability to write and further enhance their self-confidence.

Some studies have found that the establishment of students’ academic confidence is influenced by the importance of mentoring (Gearity and Mertz, 2012). The achievement motivation and active learning used in this study were based on using the students’ self-assessments as the main influence. This study was not concerned with whether external factors would affect students’ confidence building, which was a limitation of this study. In addition, this study only investigated the relationship among achievement motivation, active learning, and academic confidence but did not address the students’ background variables, such as intelligence, gender, and family economics. It is suggested that future researchers expand the study population to include different background variables so as to create different models or use background variables as control variables.

### Recommendations

The dissertation/ thesis is the last hurdle before a graduate student graduates, and it is also the hurdle that causes the most attrition of graduate students. A dissertation is a paper that a graduate student must produce within a few months, and it includes a number of processes, such as problem identification and validation, literature collection, data collection and analysis, and writing. If a graduate student fails to complete the dissertation on time, he or she will be required to delay graduation for one or more semesters (Dupont et al., 2013). Komarraju et al. (2009) suggested that motivation affects learners’ interest, emotion, and confidence in learning tasks, while CVTAE theory holds that controlling the learner’s beliefs is an essential factor, and that whether the learner will take action on a learning task depends on whether he or she thinks the task can be completed (Pekrun, 2006, 2009). Similar to the concept of self-efficacy proposed by Bandura (1997), in this study, the feasibility of a task was found to be judged by the learner before action, after which the learner’s judgment of the feasibility becomes a critical factor. Therefore, this study confirmed that the higher the motivation of graduate students to achieve, the higher the willingness to take the initiative to learn and the higher the academic confidence will be. Graduate students can gain a sense of accomplishment by setting goals, such as completing scale development or searching classical literature, and gradually increase their proportion of active learning through the guidance of their supervising professors, and eventually conduct independent research and gain academic confidence in research.

Sharma (2018) analyzed research related to stress and found that most of the studies believe that stress has negative effects on learners; however, the studies confirmed that an appropriate level of stress is beneficial for enhancing the achievement motivation of learners. It is suggested that subsequent studies include stress and investigate whether stress interacts differently with achievement motivation, active learning, and academic confidence.

In addition, Arsenis and Flores (2021) suggested that learner confidence has a critical influence on learning and achievement performance. As learners’ confidence in their learning ability affects their perceived performance on learning tasks, it is suggested that future research include learning achievement to investigate whether academic confidence predicts learning performance.

Gearity and Mertz (2012) noted that if graduate students want to find a direction for their research, they must first find supervising professors who can help them. Under the professor’s guidance, they must find the direction, questions, and structure of their research, and the professors must supervise the students to complete their research. Welton et al. (2015) confirmed that the supervising professor’s supervisory style or interaction with the graduate student also affects the completion of the graduate student’s thesis. The failure of graduate students to graduate is not only influenced by personal factors, but also by external factors. The results of this study showed that academic motivation, active learning, and academic self-confidence are positively influenced, but it is worthwhile to investigate whether the influence of external factors (e.g., Advisors) will change their influence.

## Data Availability Statement

The raw data supporting the conclusions of this article will be made available by the authors, without undue reservation.

## Ethics Statement

Ethical review and approval were not required for the study on human participants in accordance with the local legislation and institutional requirements. Written informed consent for participation was not required for this study in accordance with the national legislation and the institutional requirements.

## Author Contributions

J-CC and J-NY: concept and design and drafting of the manuscript. Y-TW and J-NY: acquisition of data and statistical analysis. J-CC and Y-TW: critical revision of the manuscript. All authors contributed to the article and approved the submitted version.

## Funding

This study was partially funded by the Ministry of Science and Technology of Taiwan, with grant number MOST 110-2511-H-027-001.

## Conflict of Interest

The authors declare that the research was conducted in the absence of any commercial or financial relationships that could be construed as a potential conflict of interest.

## Publisher’s Note

All claims expressed in this article are solely those of the authors and do not necessarily represent those of their affiliated organizations, or those of the publisher, the editors and the reviewers. Any product that may be evaluated in this article, or claim that may be made by its manufacturer, is not guaranteed or endorsed by the publisher.
